# Three-dimensional ordered mesoporous Co_3_O_4_ enhanced by Pd for oxygen evolution reaction

**DOI:** 10.1038/srep41542

**Published:** 2017-01-30

**Authors:** Qing Qu, Jian-Hua Zhang, Jing Wang, Qing-Yu Li, Chang-Wei Xu, Xihong Lu

**Affiliations:** 1 Guangzhou Key Laboratory for Environmentally Functional Materials and Technology, School of Chemistry and Chemical Engineering, Guangzhou University, Guangzhou 51006, China; 2 Guangxi Key Laboratory of Low Carbon Energy Materials, School of Chemistry and Pharmaceutical Sciences, Guangxi Normal University, Guilin, 541004, China; 3 MOE of the Key Laboratory of Bioinorganic and Synthetic Chemistry, School of Chemistry and Chemical Engineering, Sun Yat-Sen University, Guangzhou 510275, China

## Abstract

Considerable efforts have been devoted recently to design and fabrication of high performance and low cost electrocatalysts for oxygen evolution reaction (OER). However, catalytic activity of current electrocatalysts is usually restricted by high onset potential and limited active sites. Herein, we fabricated three-dimensional (3D) highly ordered mesoporous Pd-Co_3_O_4_ composite materials as excellent electrocatalysts for OER in alkaline solution with high activity and stability. Three-dimensional highly ordered mesoporous Co_3_O_4_ material was firstly synthesized using mesoporous silica KIT-6 as hard template. Then, Pd-Co_3_O_4_ nanomaterials were prepared by a simple reduction method. The as-prepared 3D mesoporous Pd-Co_3_O_4_ catalysts have ordered mesoporous structure with a high surface area of 81.0 m^2^ g^−1^. Three-dimensional highly ordered mesoporous structure can facilitate diffusion and penetration of electrolyte and oxygen. Moreover, the catalysts can also keep catalyst particles in a well dispersed condition with more catalytic active sites. Electrochemical measurements reveal that the 3D mesoporous Pd-Co_3_O_4_ catalysts exhibit superior performance in alkaline solution with low onset potential (0.415 V *vs*. SCE) and excellent long-duration cycling stability.

Catalytic splitting of water (2H_2_O → O_2_ + 2H_2_) into hydrogen and oxygen provides a potential path to product clean H_2_ and O_2_ for human society^1^,^2^. However, one of major hurdles of water electrolysis is anodic oxygen evolution reaction (OER) which needs high onset potential and shows slow sluggish kinetics due to four-electron transfer process^3^,^4^,^5^. Consequently, extensive efforts have been undertaken to develop highly efficient catalysts with low onset potential and promoted reaction kinetics^6^. Rutile type oxides RuO_2_ and IrO_2_ have been proven to be highly efficient OER catalysts^5^. Unfortunately, these noble metal oxide catalysts suffer from poor chemical stability in alkaline media and high price, which limit their practical large-scale application as water splitting anodes. Therefore, design and development of OER catalysts with low cost and high activity has attracted considerable attention, and lots of efforts have been made^7^,^8^,^9^,^10^,^11^,^12^.

Among all candidates, cobalt oxide nanoparticles have been widely explored as effective OER catalysts because of their nontoxic, earth-abundant and stable property^7^,^8^,^9^,^10^,^11^. For example, Co_3_O_4_ with different morphologies, such as hollow fluffy cage, mesoporous nanoflake, ultrathin porous nano-plate, has been used as efficient OER catalyst^13^,^14^. Additionally, 3D nanostructures are beneficial for promoting electrochemical performance of electrodes due to their interconnected pores, large specific surface area, controllable pore size and pore wall composition^15^,^16^. Template method has been considered as one of the most convenient and effective methods to prepare 3D nanostructures. Mesoporous silica, emerged as a general template for development of 3D mesoporous materials, has been widely applied to produce metal oxides such as WO_3_, CeO_2_, NiMoO_4_, Cr_2_O_3_ and Co_3_O_4_^17^,^18^,^19^,^20^,^21^,^22^,^23^.

Recently, it has been demonstrated that formation of Co(IV) cations in the cobalt oxides is a crucial step for the OER by an *ex*-*situ* electron paramagnetic resonance spectroscopy^12^,^24^. Co(IV) cations are involved as intermediate states or mediator sites, which will coordinate with OH and/or other O species and accelerate generation of oxygen at reaction interphase in the OER. Consequently, preparation of catalysts with high concentration of Co(IV) cations is of great importance. In the past decade, noble metals, with strong electron inductive effect, have been widely used as electron adsorbates and active sites to facilitate deprotonation of higher value oxide species^25^,^26^,^27^,^28^,^29^,^30^,^31^,^32^,^33^. Yeo *et al*. have deposited monolayer cobalt oxide on Au, which showed OER activity of 40 times as high as bulk cobalt oxide, and nearly 3 times as high as bulk Ir^34^. Thus high OER activity can be attributed to increased fraction of the Co(IV) cations. In compared with Au, Pd has much higher conductivity and has shown substantially higher catalytic activity for OER^35^.

Herein, we synthesized 3D mesoporous Co_3_O_4_ materials with high surface area using KIT-6 as hard template. Then, optimized Pd nanoparticles were dispersed onto the 3D mesoporous Co_3_O_4_ nanostructures (Pd-Co_3_O_4_) in order to create Co_3_O_4_ catalysts with high concentration of Co(IV) cations. The 3D mesoporous Co_3_O_4_ and Pd-Co_3_O_4_ were applied as electrocatalysts for OER, and demonstrated to have outstanding electrochemical performance, which are compared with common Co_3_O_4_/C material. This development will broaden our horizon for design and application of 3D mesoporous nanostructure catalysts in energy and environment areas.

## Results

**Figure 1:**
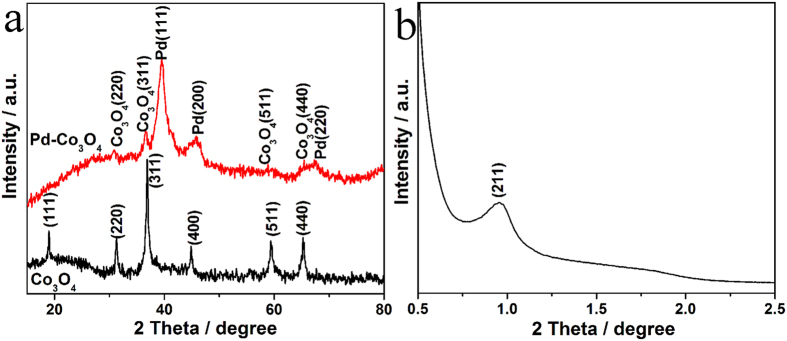
(**a**) XRD patterns for mesoporous Co_3_O_4_ and Pd-Co_3_O_4_ (wt 1:1) catalysts; (**b**) low-angle XRD pattern for mesoporous Co_3_O_4_.

As shown in Fig. 1a, X-ray diffraction (XRD) pattern of mesoporous Co_3_O_4_ exhibits diffraction peaks at 19.0°, 31.2°, 36.9°, 44.8°, 59.4° and 65.2°, which are assigned to (111), (220), (311), (400), (511) and (440) facets of cubic crystallite Co_3_O_4_ (JPCD No. 43–1003, space group Fm3m). This result indicates that cobalt precursor has been completely transformed into crystalline Co_3_O_4_ using nanocasting from mesoporous silica as hard template. Structure regularity of mesoporous Co_3_O_4_ was examined by small angle XRD as shown in Fig. 1b. One relatively sharp diffraction peak at 0.9° was observed which can be indexed as (211) reflection in the cubic Ia3d space group. This confirms that the mesoporous Co_3_O_4_ materials remain highly ordered structure of the cubic Ia3d symmetry originated from the silica template^37^,^38^. After being decorated with Pd, there are some additional strong diffraction peaks for Pd-Co_3_O_4_ (wt 1:1) at 39.5°, 45.7° and 67.4°, which correspond to (111), (200) and (220) facets of metallic Pd. All of the diffraction peaks of Pd and Co_3_O_4_ are observed, indicating successfully synthesis of mesoporous Pd-Co_3_O_4_ catalyst.

**Figure 2:**
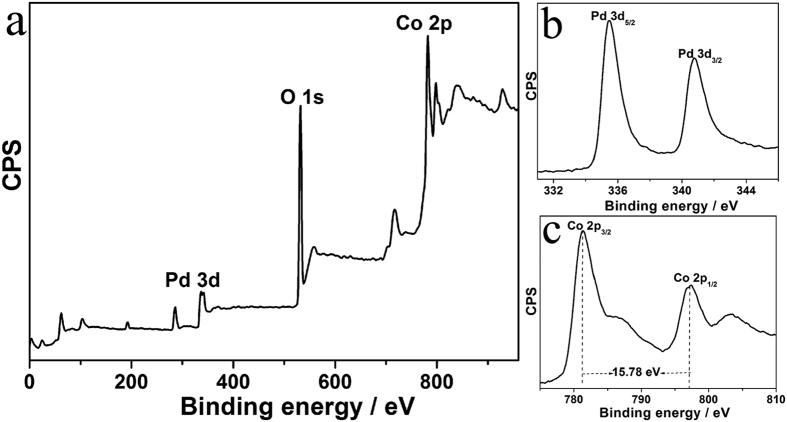
XPS spectra (**a**) overall, (**b**) Pd 3d and (**c**) Co 2p of mesoporous Co_3_O_4_ and Pd-Co_3_O_4_ (wt 1:1).

In order to investigate composition of the Pd-Co_3_O_4_(wt 1:1) catalyst, X-ray photoelectron spectroscopy (XPS) analyses were further performed. Figure 2a displays XPS survey spectra, where Pd, O and Co signals are observed besides C signals. Other signals have not been discovered, which reveal that no SiO_2_ remains after soaking in NaOH solution. Two peaks are centred at 335.44 and 340.78 eV, which could be attributed to metallic Pd^0^ (Fig. 2b)^39^. As shown in Fig. 2c, values of binding energy of Co 2p_1/2_ and Co 2p_3/2_ in the Pd-Co_3_O_4_ (wt 1:1) are 781.37 and 797.15 eV, which are in accord with values for the characteristic Co 2p peaks of Co_3_O_4_. A spin–orbit splitting energy between Co 2p_1/2_ and Co 2p_3/2_ is 15.78 eV, which is apparently different from that of Co 2p of CoO (16.00 eV) and Co_2_O_3_ (15.00 eV)^40^. This result further indicates that the as-prepared cobalt oxide is mixed-valence Co_3_O_4_.

**Figure 3:**
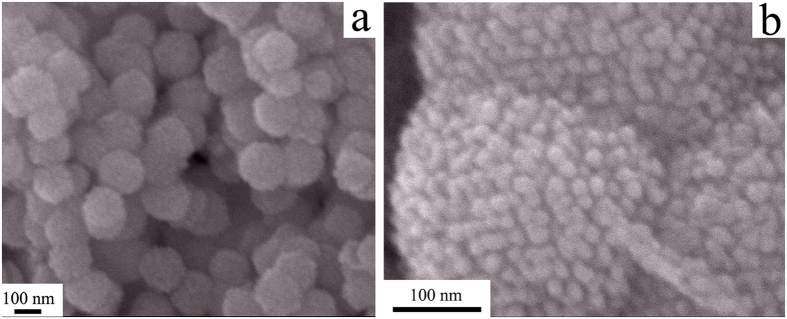
SEM images for mesoporous Co_3_O_4_.

**Figure 4:**
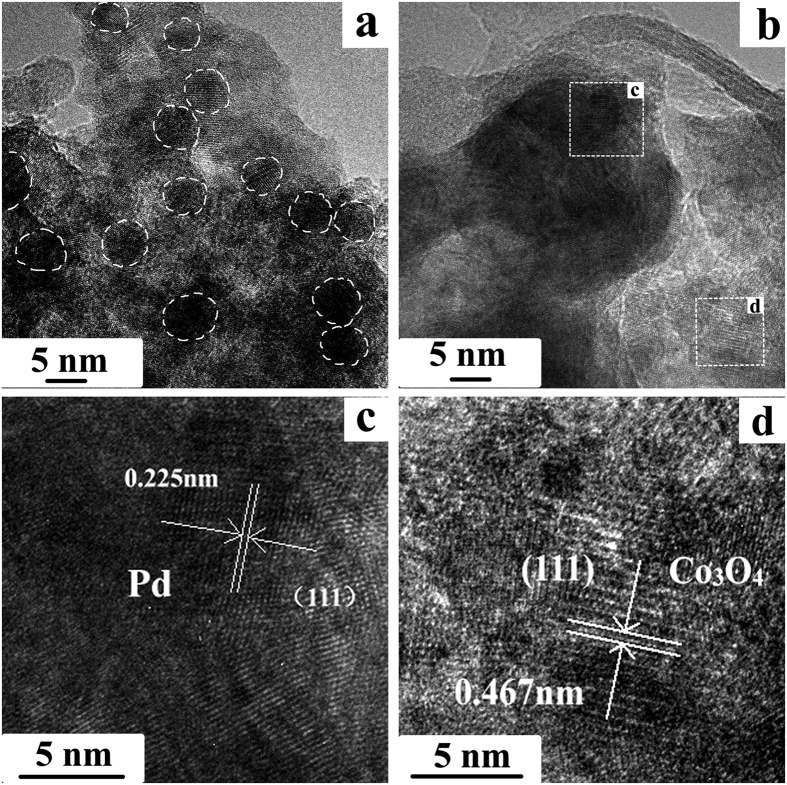
(**a**,**b**) TEM images of Pd-Co_3_O_4_(wt 1:1) catalyst; (**c**,**d**) enlarged images of white rectangles marked in (**b**).

Scanning electron microscopy (SEM) images in Fig. 3 clearly reveal that microsphere particle size of as-prepared well-ordered mesoporous Co_3_O_4_ is ranging from 200 to 250 nm with high uniformity. The mesoporous nanostructure is made up of plenty of narrow gaps of each small Co_3_O_4_ particle with adjacent particles with a diameter of ~20 nm (Fig. 3b). In order to study microstructure of Pd-Co_3_O_4_ (wt 1:1), further transmission electron microscopy (TEM) analyses were conducted. Representative TEM images are depicted in Fig. 4a,b, showing that Pd particles presented as darker spots with white line of dash to emphasize in a diameter of around 5–8 nm are in unified dispersion into the Co_3_O_4_ matrix in Fig. 4a. As shown in Fig. 4c, an approximate well lattice spacing in the HR-TEM image is 0.225 nm, which originates from (111) plane of Pd. A parallel fringe with a spacing of 0.467 nm is in correspondance to (111) plane of cubic Co_3_O_4_ (Fig. 4d), which is in accordance with the XRD result. These results confirm that the Pd-Co_3_O_4_ electrocatalysts are composed of small Pd particles embed into the Co_3_O_4_ structure.

**Figure 5:**
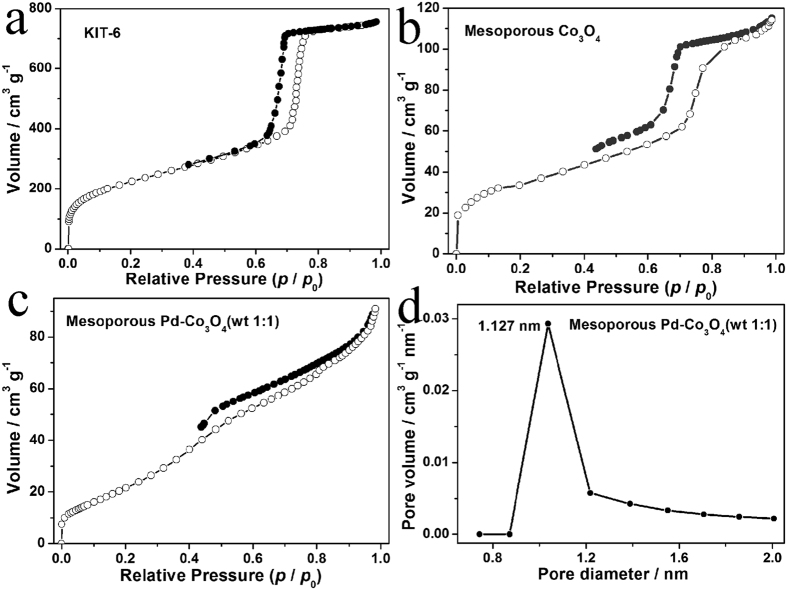
Nitrogen adsorption and desorption isotherms of (**a**) KIT-6, (**b**) mesoporous Co_3_O_4_, (**c**) Pd-Co_3_O_4_(wt 1:1); (**d**) pore size distribution of Pd-Co_3_O_4_(wt 1:1).

Nitrogen adsorption-desorption isotherm and corresponding pore size distribution have been carried out to confirm the mesoporous nature of the KIT-6, mesoporous Co_3_O_4_ and Pd-Co_3_O_4_ (wt 1:1). As shown in Fig. 5a–c, curves of all the samples demonstrate a type-IV isotherm with a H3 hysteresis loop in a *p/p*_0_ range from 0.4 to 1, which is characteristic of mesoporous materials^41^. A calculated BET surface area of KIT-6 is 817.9 m^2^ g^−1^. However, surface area of mesoporous Co_3_O_4_ sharp decreases to 134.0 m^2^ g^−1^, yet still is higher than that of previously reported ordered mesoporous Co_3_O_4_ as shown in Table 1 9, indicating that the Co_3_O_4_ is filled into the pore space of KIT-6. After loading Pd nanoparticles, surface area of the Pd-Co_3_O_4_ (wt 1:1) decreases slightly to 81.0 m^2^ g^−1^, also is higher than 70.5 m^2^ g^−1^ of rhombus-shaped Zn/Ni-doped Co_3_O_4_^42^. The pore size distribution of mesoporous Pd-Co_3_O_4_ (wt 1:1) calculated from BJH method is shown in Fig. 5d. A peak at mean value demonstrates a centralized pore-size distribution ranging from 0.5 to 2.0 nm, further confirming coexistence of mesopores in the material. The mesoporous Pd-Co_3_O_4_ (wt 1:1) with 1.127 nm of mode pore diameter has the highest pore volume about 0.03 cm^3^ g^−1 ^nm^−1^. This result indicates that Pd nanoparticles might have no big influence on effective surface area contact with electrolyte.

**Figure 6:**
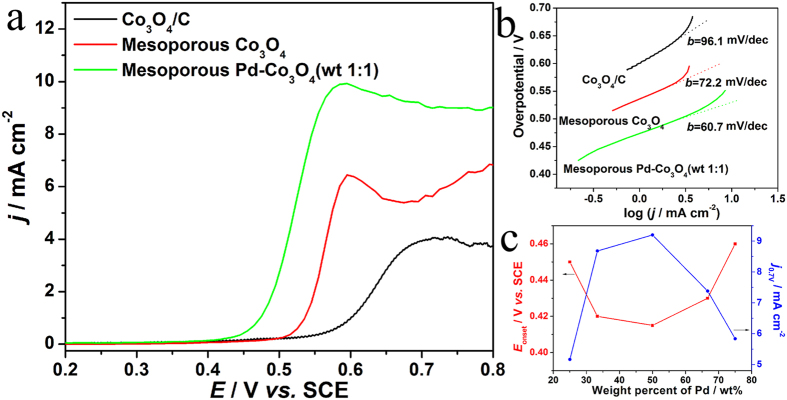
(**a**) LSV curves in 0.1 mol L^−1^ KOH with a sweep rate of 0.001 V s^−1^ on Co_3_O_4_/C, mesoporous Co_3_O_4_ and Pd-Co_3_O_4_ (wt 1:1) electrodes; (**b**) Tafel plots in 0.1 mol L^−1^ KOH with a sweep rate of 0.001 V s^−1^ on Co_3_O_4_/C, mesoporous Co_3_O_4_ and Pd-Co_3_O_4_ (wt 1:1) electrodes; (**c**) Plots of *E*_onset_ and *j*_0.7V_ in LSV curves as a function of the Au weight percent in mesoporous Pd-Co_3_O_4_ with a total loading of 0.1 mg cm^−2^ on the electrodes.

For illustrate of superior water splitting performance of mesoporous Co_3_O_4_ electrocatalyst, OER activity of mesoporous Co_3_O_4_ is investigated through linear sweep voltammetry (LSV) in 0.1 mol L^−1^ KOH with a sweep rate of 1 mV s^−1^. As the reaction is proceeding, oxygen bubbles coalesce and block active sites on the electrode surface arousing fluctuation of the curves, especially after 0.58 V. When the potential on the electrodes increases, the bubbles coalesce and evolve vacating the sites which occupy on the surface. As shown in Fig. 6a, current density at 0.7 V (*j*_0.7V_) on the mesoporous Co_3_O_4_ electrode is 5.5 mA cm^−2^, 1.4 times as high as that on previous reported Co_3_O_4_/C electrode (3.9 mA cm^−2^). Moreover, the mesoporous Co_3_O_4_ electrode shows an onset potential (*E*_onset_) of 0.508 V, much lower than Co_3_O_4_/C electrode (0.545 V)^32^. This performance is also comparable to the best performance of reported Co_3_O_4_ nanoflake and rhombus-shaped Co_3_O_4_, 16.4 and 90 mV separately lower towards the OER under the same condition as shown in Table 1^13^,^43^. This improvement can attribute to high surface area of 3D ordered mesoporous structure, which can provide more active sites for facilitate of charge transfer at nano-scale Co_3_O_4_ walls/electrolyte interface. After loading Pd nanoparticles on mesoporous Co_3_O_4_, *E*_onset_ value of mesoporous Pd-Co_3_O_4_ (wt 1:1) electrode is 0.415 V, 65 mV shifting negatively compared with mesoporous Co_3_O_4_ electrode, and 130 and 115 mV separately to previously rhombus-shaped Zn/Ni-doped Co_3_O_4_ and mesoporous Fe-Co_3_O_4_^42^,^43^. Moreover, value of *j*_0.7V_ on the mesoporous Pd-Co_3_O_4_ (wt 1:1) electrode is 9.2 mA cm^−2^, 1.6 times as high as that on the mesoporous Co_3_O_4_ electrode. This substantially higher electrocatalytic activity of the Pd-Co_3_O_4_ (wt 1:1) electrode presents a synergistic effect between Pd and Co_3_O_4_. Tafel plots for OER activity on the electrodes are presented in Fig. 6b. The Tafel values on the Co_3_O_4_/C and mesoporous Co_3_O_4_ electrodes are 96.1 and 72.2 mV dec^−1^. Tafel value on the mesoporous Co_3_O_4_ electrode is lower than that on the Co_3_O_4_/C electrode, which indicates that OER occurs favourably on the mesoporous Co_3_O_4_. Tafel value on the mesoporous Pd-Co_3_O_4_ (wt 1:1) electrode is 60.7 mV dec^−1^, lower than that on the mesoporous Co_3_O_4_ electrode, representing that Pd addition promotes activity of OER on the mesoporous Co_3_O_4_. For further exploration effect of Pd mass on electrochemical activity, mesoporous Pd-Co_3_O_4_ catalysts with various ratios of Pd and Co_3_O_4_ have been synthesized and investigated by similar method. Ratio of Pd and Co_3_O_4_ was determined by ICP-OES (PerkinElmer, USA). Figure 6c compares the values of *E*_onset_ and *j*_0.7V_ of mesoporous Pd-Co_3_O_4_ catalysts with different Pd weight percentages. The lowest onset potential of these catalysts is 0.415 V. The current density at 0.7 V vs. SCE and Pd content is in positive correlation until later reaches a maximum value (50 wt% Pd). Therefore, 50 wt% proves to be the best weight ratio for Pd in the Pd-Co_3_O_4_ material with the lowest onset potential and largest current density.

**Figure 7:**
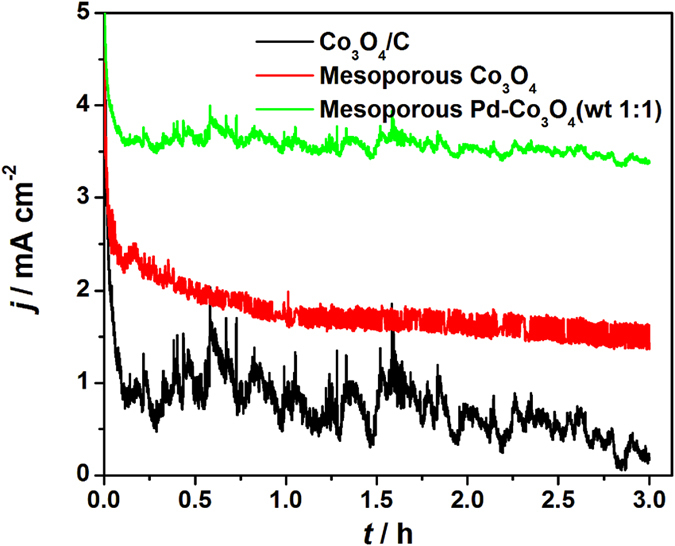
Chronoamperometry curves on Co_3_O_4_/C, mesoporous Co_3_O_4_ and Pd-Co_3_O_4_ (wt 1:1) electrodes in 0.1 mol L^−1^ KOH at potential of 0.7 V.

Long-term chronoamperometry curves (*i*-*t* curves) on the Co_3_O_4_/C, 3D mesoporous Co_3_O_4_ and Pd-Co_3_O_4_(wt 1:1) catalysts for OER were collected at 0.7 V for 3 h in 0.1 mol L^−1^ KOH solution, as shown in Fig. 7. The mesoporous Co_3_O_4_ and Pd-Co_3_O_4_ (wt 1:1) electrodes show excellent durability in contrast to a sharp activity loss of Co_3_O_4_/C. At end of long-term experiment, the mesoporous Pd-Co_3_O_4_ (wt 1:1) electrode achieves an oxidation current density of 3.4 mA cm^−2^, which is 2.3 times as higher as that on the mesoporous Co_3_O_4_ electrode (1.5 mA cm^−2^) and 3.8 times as higher as that on the Co_3_O_4_/C (0.4 mA cm^−2^). This stability can be attributed to mesoporous structure which can keep the catalyst particles in a well dispersed condition with more catalytic active sites. During the long-term OER test, small oxygen bubbles will coalesce on the surface of electrode and block contact between electrolyte and surface active sites of the catalyst, resulting in slowly decrease of oxidation current density. Then, the oxygen bubbles grow larger slowly and release from the electrode, renewing the contact between electrolyte and electrode. The oxygen generation and release produce perturbation and ‘current waves’ in long-term *i*-*t* curves. However, perturbation of the 3D mesoporous Co_3_O_4_ and Pd-Co_3_O_4_ (wt 1:1) electrodes is much smaller than that of Co_3_O_4_/C electrode, showing that the 3D mesoporous structure is also benefit for migration and release of oxygen gas.

**Figure 8:**
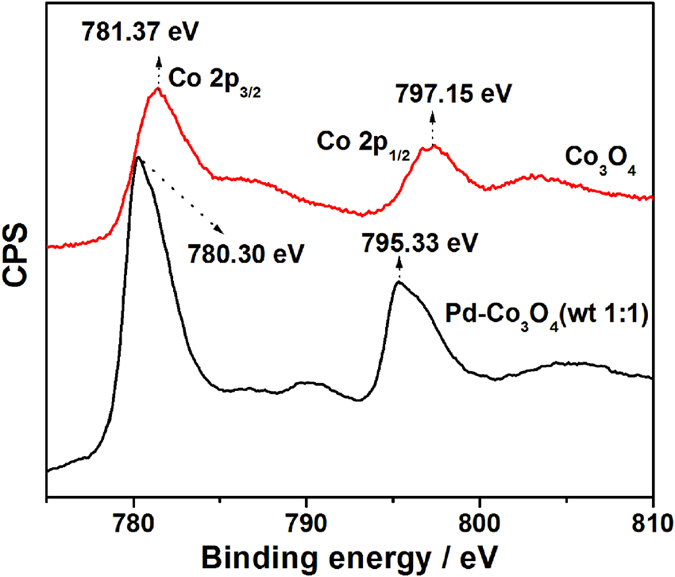
XPS spectra of Co 2p for mesoporous Co_3_O_4_ and mesoporous Pd-Co_3_O_4_ (wt 1:1).

To gain insight into the prominent OER activity of 3D mesoporous Pd-Co_3_O_4_ (wt 1:1) catalyst, XPS Co 2p core levels of 3D mesoporous Co_3_O_4_ and Pd-Co_3_O_4_ (wt 1:1) catalysts are compared (Fig. 8). Compared to Co 2p_3/2_ peak of Co_3_O_4_, positive shift around 1.07 eV in binding energy is observed in Co2p_3/2_ peak of the Pd-Co_3_O_4_ (wt 1:1) catalyst, which means that more Co(IV) species are generated after introducing Pd. As discussed above, metallic Pd is a highly electronegative metal and can acts as an electron adsorbate. After Pd nanoparticles embed into the mesoporous Co_3_O_4_ structure, electrons in Co(III) species will migrate to Pd, leading to higher oxidation states of Co(IV). Presence of strong electrophilic Co(IV) species can accelerate formation of OOH species *via* nucleophilic reaction with OH and other O species^44^. Depending on electrochemical oxidation, progressive oxidation from Co(III) to Co(IV) is supposed as rate-limiting step, so increased amount of Co(IV) cations results in enhanced OER performance. Therefore, the 3D mesoporous Pd-Co_3_O_4_ (wt 1:1) materials show superior OER activity than 3D mesoporous Co_3_O_4_.

In conclusion, (3D) highly ordered mesoporous Pd-Co_3_O_4_ composite materials show high activity and stability as excellent electrocatalysts for OER in alkaline solution prepared by mesoporous silica KIT-6 as hard template. Thus 3D highly ordered mesoporous structure can facilitate diffusion and penetration of electrolyte and oxygen. Moreover, it can also keep the catalyst nanoparticles in a well dispersed condition with more catalytic active sites. The as-prepared mesoporous Co_3_O_4_ has an ordered Ia3d symmetric mesoporous structure with a high surface area of 134 m^2^ g^−1^, while the 3D mesoporous Pd-Co_3_O_4_ catalysts also have a high surface area of 81.0 m^2^ g^−1^. Onset potential of mesoporous Pd-Co_3_O_4_(wt 1:1) electrode is 0.415 V, which shifts negatively 65 mV compared with mesoporous Co_3_O_4_ electrode. Moreover, the value of *j*_0.7V_ on the mesoporous Pd-Co_3_O_4_(wt 1:1) electrode is 9.2 mA cm^−2^, which is 1.6 times as high as that on the mesoporous Co_3_O_4_ electrode. Such outstanding electrocatalytic activity is attributed to the higher oxidation state of Co(IV) species in the Pd-Co_3_O_4_ catalysts by introducing metallic Pd nanoparticles. This present development will broaden our horizon for design and applications of 3D mesoporous catalysts in energy and environment areas.

## Methods

### Materials synthesis

Expect poly(ethyleneglycol)-block-poly (propylene glycol)-block-poly (ethylene glycol) surfactant (Pluronic 123 or P123), all chemicals were purchased from Aladdin and used as received. The hard template, highly ordered mesoporous SiO_2_ (KIT-6) materials were synthesized according to the previously report^36^. Typically, 10 g P123 was dissolved in a mixed solution of 360 g distilled water, 21.5 g concentrated HCl (32%) and 10 g n-butanol under stirring at 308 K. One hour later, 21.5 g tetraethoxysilane (TEOS) was added to the above solution with another 24 h strong stirring. Then, the mixture was transferred into a closed Teflon-lined stainless steel autoclave and heated at 373 K for 24 h. Later, the white solid power was filtered, following by washed with ethanol-HCl mixture and dried at 373 K. Finally, the solid power sintered at 323 K for 6 h to remove surfactant to obtain final mesoporous SiO_2_ (KIT-6). The mesoporous Co_3_O_4_ replica from KIT-6 was obtained by a nanocasting method. Generally, 0.4 g KIT-6 and 3–6 mmol Co(NO_3_)_3_·6H_2_O were dispersed into 20–30 mL ethanol. After being stirred for 12 h, the ethanol was removed by evaporation at room temperature. Then, the solid material was sintered at 673 K for 2 h in order to decompose the nitrate. At last, the KIT-6 template was removed by soaking in 2 mol L^−1^ NaOH solution for 12 h with strong stirring at 363 K, followed by being washed with deionized water and dried at 323 K. The mesoporous Pd-Co_3_O_4_ nanomaterials were prepared by reduction of Pd(NH_3_)_4_Cl_2_ solutions with adding excess of 0.01 mol L^−1^ NaBH_4_ solution. After the mesoporous Co_3_O_4_ powder synthesized, it was put into distilled water with corresponding mass ratio and mixed with Pd(NH_3_)_4_Cl_2_ solution according to different certain proportion. What is noteworthy that Pd(NH_3_)_4_Cl_2_ solution was added in drops, and time for the drop space is ten minutes approximately. When Pd(NH_3_)_4_Cl_2_ solution was exhausted, the homogeneous mixture was standing at least 8 hours to ensure Pd nanoparticles attaching with the surface on mesoporous Co_3_O_4_ to be reduced completely. Ratio of Pd and Co_3_O_4_ can be adjusted by adding different amount of Pd(NH_3_)_4_Cl_2_. The mesoporous Co_3_O_4_ and Pd-Co_3_O_4_ power were dispersed in deionized water with 5 wt% PTFE under ultrasonic stirring. Then, the catalyst ink was deposited on surface of a graphite rod with a geometric area of 0.33 cm^2^ and dried at 263 K for 30 min. Loading of carbon black and PTFE on the electrodes was accurately controlled at 0.23 and 0.1 mg cm^−2^. Total loading of amount of Pd and Co_3_O_4_ in the catalysts on electrodes was accurately controlled at 0.1 mg cm^−2^.

### Characteriazation

XRD was carried out using a Panalytical X’Pert powder X-ray diffractometer with Cu Kα radiation (*λ* = 0.15418 nm). SEM images were obtained using a Quanta 400 FEG microscope (FEI Company). TEM images were carried out on a JEOL JEM-2010 (JEOL Ltd.). XPS measurements were performed in an ESCALAB 250 spectrometer. The ratio of Pd and Co_3_O_4_ was tested by ICP-OES (PerkinElmer, USA). Nitrogen adsorption isotherms were measured with a Beckman Coulter sorption analysis at 77 K in liquid nitrogen. Prior to measurements, the samples were degassed at 473 K for 10 h. Brunauer-Emmett-Teller (BET) surface area was calculated using experimented points at a relative pressure of *p/p*_0_ = 0.05–0.25. Pore size distribution (PSD) curve was calculated by the BJH (Barrett-Joyner-Halenda) method from desorption branch. Total pore volume was estimated by nitrogen amount adsorbed at a relative pressure (*p/p*_0_) of 0.99. All electrochemical measurements were carried out in 0.1 mol L^−1^ KOH solution using a standard three-electrode cell at 298 K by Solartron 1287. A platinum foil (3.0 cm^2^) was used as counter electrode, while a saturated calomel electrode with a salt bridge (SCE, 0.241 V versus SHE) was used as reference electrode.

## Additional Information

**How to cite this article:** Qu, Q. *et al*. Three-dimensional ordered mesoporous Co_3_O_4_ enhanced by Pd for oxygen evolution reaction. *Sci. Rep.* **7**, 41542; doi: 10.1038/srep41542 (2017).

**Publisher's note:** Springer Nature remains neutral with regard to jurisdictional claims in published maps and institutional affiliations.

## Figures and Tables

**Table 1:** Comparison of mesoporous Co_3_O_4_ and metal-doped Co_3_O_4_ catalysts.

	BET surface area/(m^2^ g^−1^)	*E*_onset_/V
mesoporous Co_3_O_4_ (this work)	134.0	0.508
^9^mesoporous Co_3_O_4_	108.6	
^42^rhombus-shaped Co_3_O_4_		0.598
^13^nanoflake Co_3_O_4_		0.524
mesoporous Pd-Co_3_O_4_ (this work)	81.0	0.415
^42^rhombus-shaped Zn/Ni-doped Co_3_O_4_	70.5	0.545
^43^mesoporous Fe-Co_3_O_4_		0.530
